# Mortality attributable to type 2 diabetes mellitus in Latin America and the Caribbean: a comparative risk assessment analysis

**DOI:** 10.1136/bmjdrc-2021-002673

**Published:** 2022-02-19

**Authors:** Wilmer Cristobal Guzman-Vilca, Rodrigo M Carrillo-Larco

**Affiliations:** 1 CRONICAS Centre of Excellence in Chronic Diseases, Universidad Peruana Cayetano Heredia, Lima, Peru; 2 Sociedad Científica de Estudiantes de Medicina Cayetano Heredia (SOCEMCH), Universidad Peruana Cayetano Heredia, Lima, Peru; 3 Department of Epidemiology and Biostatistics, School of Public Health, Imperial College London, London, UK; 4 Universidad Continental, Lima, Peru

**Keywords:** population health, risk assessment

## Abstract

**Introduction:**

We quantified the proportion and the absolute number of deaths attributable to type 2 diabetes mellitus (T2DM) in Latin America and the Caribbean (LAC) using an estimation approach.

**Research design and methods:**

We combined T2DM prevalence estimates from the NCD Risk Factor Collaboration, relative risks between T2DM and all-cause mortality from a meta-analysis of cohorts in LAC, and death rates from the Global Burden of Disease Study 2019. We estimated population-attributable fractions (PAFs) and computed the absolute number of attributable deaths in 1990 and 2019 by multiplying the PAFs by the total deaths in each country, year, sex, and 5-year age group.

**Results:**

Between 1985 and 2014 in LAC, the proportion of all-cause mortality attributable to T2DM increased from 12.2% to 16.9% in men and from 14.5% to 19.3% in women. In 2019, the absolute number of deaths attributable to T2DM was 349 787 in men and 330 414 in women. The highest death rates (deaths per 100 000 people) in 2019 were in Saint Kitts and Nevis (325 in men, 229 in women), Guyana (313 in men, 272 in women), and Haiti (269 in men, 265 in women).

**Conclusions:**

A substantial burden of all deaths is attributed to T2DM in LAC. To decrease the mortality attributable to T2DM in LAC, policies are needed to strengthen early diagnosis and management, along with the prevention of complications.

**Significance of this study**

**What is already known about this subject?**

- Current estimates of deaths attributable to type 2 diabetes mellitus (T2DM) in Latin America and the Caribbean (LAC) are based on risk data retrieved from non-LAC populations, which may not represent the LAC epidemiological scenario.

**What are the new findings?**

- All LAC countries increased their proportion of all-cause mortality attributable to T2DM in the last 30 years (by ~4.7% in men and ~4.8% in women).
- Most LAC countries increased their death rates (eg, some even doubled their 1990 death rate).
- LAC countries where gross domestic product per capita increased the most from 1990 to 2019 reduced their T2DM-attributable death rates throughout the same period.

**How might these results change the focus of research or clinical practice?**

- These findings call for urgent actions in LAC to reduce the mortality burden associated with T2DM.

## Introduction

Being one of the global leading causes of morbidity, mortality, and disability, type 2 diabetes mellitus (T2DM) is a global health issue.^1^ With a growing number of cases globally,^2^ disproportionally affecting low- and middle-income countries like those in Latin America and the Caribbean (LAC),^3^ a thorough quantification of the long-term outcomes (eg, mortality) of people with T2DM is key to understand the T2DM epidemiology and to set priorities while wisely allocating resources to where most needed.

Previous studies conducted in LAC have assessed national diabetes mortality based on death certificates.^4–7^ Countries in LAC still face difficulties to meet high levels of registered deaths; even when deaths certificates are available, these may have errors, inconsistencies, or garbage codes.^8 9^ Overall, T2DM mortality based on death certificates may be underestimated.^10–12^ The estimation approach (eg, a comparative risk assessment) could provide better evidence of mortality attributable to T2DM; in fact, mortality estimates based on these methods are usually higher than those based on vital registries (VRs).^13–15^ However, evidence about mortality attributable to T2DM based on the estimation approach is scarce in LAC. In addition, global estimates for LAC have focused on high glucose levels rather than T2DM diagnosis.^16^ Moreover, these global estimates were based on risk estimates from North America, Europe, and Asia,^17^ which may not represent the epidemiological scenario in LAC.^18^

Quantifying mortality attributable to T2DM based on risk estimates from LAC could provide more accurate findings to inform policies, interventions, and guidelines. In this line, a comparable and consistent quantification of mortality attributable to T2DM in LAC could also provide evidence to assess the path towards local, regional, and international commitments including the Sustainable Development Goal target 3.4 and the Pan American Health Organization plan of action for the prevention and control of non-communicable diseases (NCDs).^19 20^ Following a comparative risk assessment approach, benefiting from relative risks from LAC cohort studies,^18^ and global T2DM prevalence and mortality estimates,^1 3 21^ we computed the absolute number of all-cause deaths attributable to T2DM in 35 countries and territories in LAC in 1990 and 2019.

## Research design and methods

### Study overview

We followed the comparative risk assessment framework to assess the burden of T2DM on all-cause mortality in LAC. Population-attributable fractions (PAFs) were computed combining country-specific T2DM prevalence estimates^3^ and relative risks (RRs) of the association between T2DM and all-cause mortality in LAC.^18^ We estimated the absolute number of T2DM-attributable deaths in 1990 and 2019 by multiplying the PAFs by the total deaths in each country, year, sex, and 5-year age group. In addition, we presented the mortality rates attributable to T2DM in relation to relevant economic metrics (gross domestic product (GDP) per capita, rurality, and human development index (HDI)).

### Data sources

#### Diabetes prevalence

The prevalence estimates of T2DM, stratified by country, sex, and 5-year age groups in adults ≥20 years were downloaded from the NCD Risk Factor Collaboration (NCD-RisC).^3 22^ NCD-RisC methods have been reported elsewhere in detail.^3^ Briefly, NCD-RisC pooled population-based studies collected diabetes prevalence data and converted these data into a common diabetes definition. Data sources analyzed by the NCD-RisC included at least one T2DM biomarker. We used estimates for 1985 and 2014 to compute the absolute number of deaths attributable to T2DM in 1990 and 2019, respectively. We assumed a 5-year lag period between exposure to T2DM and mortality; in other words, T2DM prevalence in 1985 was used to compute the PAF in 1990 and T2DM prevalence in 2014 was used to compute the PAF in 2019. Additionally, a sensitivity analysis was performed using a 10-year lag period between T2DM and mortality; lag periods >10 years were not assessed because of data availability.

#### Relative risks

RRs of all-cause mortality in people from LAC with and without T2DM were retrieved from a recent meta-analysis of cohort studies in LAC.^18^ This meta-analysis provided age-specific RRs in two age groups: 35–59 and 60–74 years old. We used interpolation^17^ to calculate RRs by 5-year age groups (online supplemental table 1). Of note, these age-specific RRs were derived from population with diagnosed T2DM (ie, self-reported diagnosis). We used the same RRs for men and women because the meta-analysis did not provide age- and sex-specific RRs.

10.1136/bmjdrc-2021-002673.supp1 Supplementary data

#### All-cause mortality

The 2019 Global Burden of Disease (GBD) estimates of deaths from all causes in adults ≥20 years of the years 1990 and 2019 were used.^8 21^ Mortality data sources used by the GBD were mostly VR reported by each country. The GBD estimates account for garbage codes (ie, codes to which deaths were allocated that should have not been considered the underlying cause of death (UCD)).

#### Population

Population data specific to each country, year, sex, and 5-year age group were obtained from the 2019 GBD as well.^21^ Attributable deaths to T2DM were expressed as death rates per 100 000 people on the basis of the WHO standard population.^23^

#### Economic metrics

The following economic metrics were used: GDP per capita, proportion of people living in rural areas, and HDI. The GDP (in 1990 and 2019) per capita in constant 2010 USD and the proportion of people living in rural areas (presented as percentage, in 1990 and 2019) were retrieved from the World Bank.^24 25^ The HDI (in 1990 and 2019) was retrieved from the United Nations Development Programme.^26^

### Statistical analysis

The PAF quantifies the fraction of all-cause deaths attributed to T2DM in each country, year, sex, and 5-year age group. We used the following equation.^14 27^

$$PAF_{a,s,c}=\frac{P_{a,s,c}(RR_{a}-1)}{P_{a,s,c}(RR_{a}-1)+1}$$

where the subscripts *a*, *s*, and *c* indicate each 5-year age group, sex, and country, *P* is the T2DM prevalence, and *RR* is the RR of mortality between populations with and without T2DM. We calculated the absolute number of deaths attributable to T2DM as the product of the country-year-sex-age-specific PAF and the total number of deaths in the same strata. All computations were conducted across 5-year age groups in each country.

We propagated the uncertainty of our data sources into our final estimates computing 1000 random draws using the mean and SD of the prevalence and mortality estimates assuming a log-normal distribution. Likewise, we computed 1000 random draws of the RR using their mean and SE. From the 1000 PAF and attributable deaths computed for each country-age-sex group, the median of the distribution was herein reported as the main result and the 2.5 and 97.5 percentiles as the 95% CI. This process is consistent with the GBD methodology.^16^

As NCD-RisC prevalence estimates accounted for total T2DM (ie, both diagnosed and undiagnosed), but the RR were derived from people with diagnosed T2DM only,^18^ a sensitivity analysis for diagnosed T2DM only was performed. We assumed that 58% of the total T2DM prevalence in each country in LAC corresponded to diagnosed T2DM.^2 28^ We multiplied the available prevalence estimates by 0.58 to approximate to the prevalence of known diabetes. We thereafter followed the same general analytical approach using these new prevalence estimates as sensitivity analysis.

We used scatter plots to correlate the T2DM-attributable death rates and economic metrics; these plots showed the Pearson correlation coefficients. These correlations were only assessed for the main analysis. We used R (V.4.0.3) for the analyses and figures. The analysis code and datasets are available as online supplemental materials.

10.1136/bmjdrc-2021-002673.supp2 Supplementary data

10.1136/bmjdrc-2021-002673.supp3 Supplementary data

### Ethics

The opinions presented in this work are those of the authors alone and do not necessarily represent those of the institutions to which they belong. The authors are collectively responsible for the accuracy of the findings presented.

## Results

**Figure 1:**
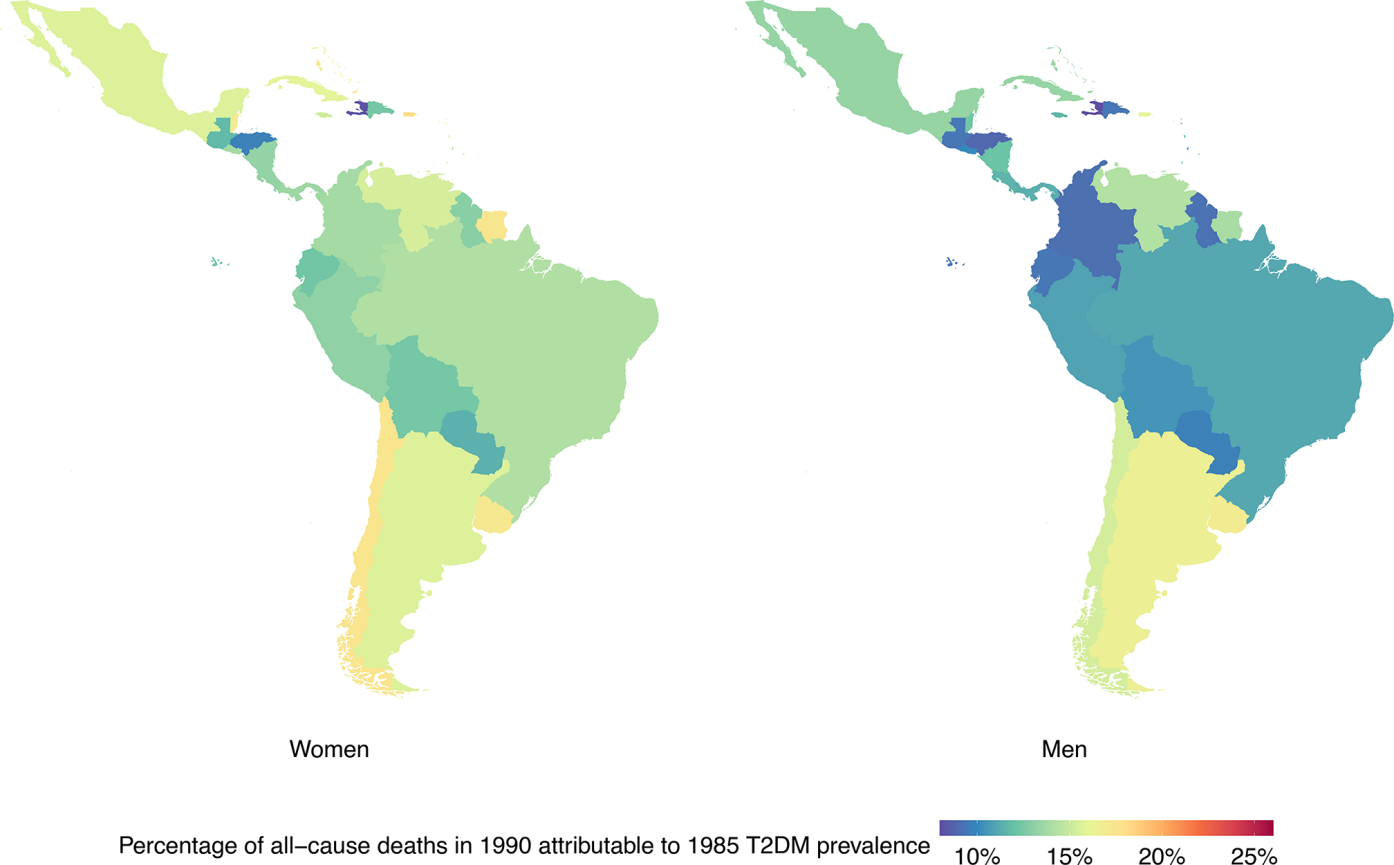
Percentage of deaths in 1990 attributable to 1985 type 2 diabetes mellitus (T2DM) prevalence by country and sex. Exact number estimates (along with their 95% CI) are presented in online supplemental table 2.

**Figure 2:**
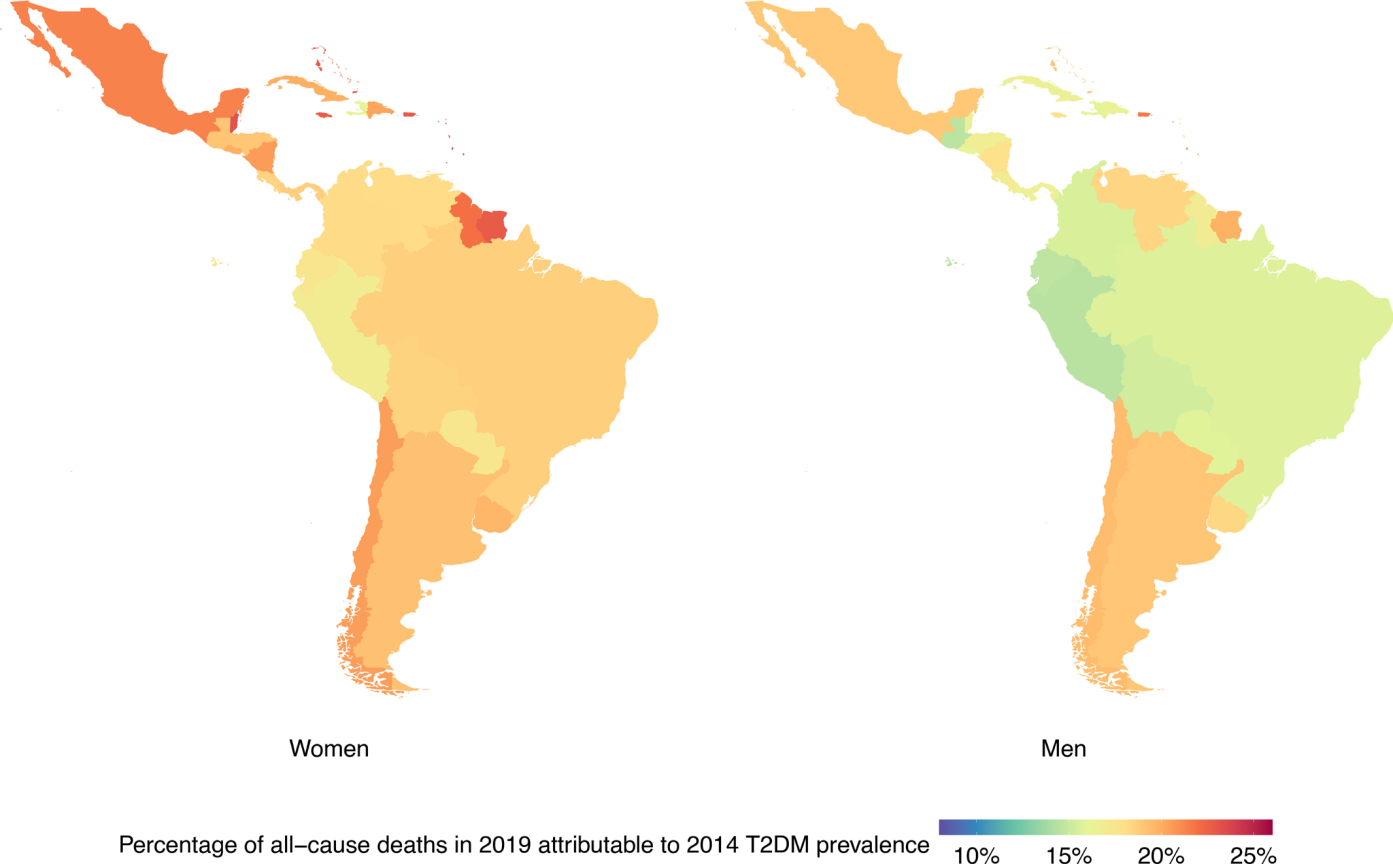
Percentage of deaths in 2019 attributable to 2014 type 2 diabetes mellitus (T2DM) prevalence by country and sex. Exact number estimates (along with their 95% CI) are presented in online supplemental table 2.

The proportion of all-cause mortality attributable to T2DM increased in all LAC countries from 1990 to 2019 (figures 1–2). Over this period, the fraction of all-cause mortality attributable to T2DM increased from 12.2% (95% CI: 4.0% to 22.7%) to 16.9% (95% CI: 6.3% to 30.3%) in men and from 14.5% (95% CI: 4.8% to 26.9%) to 19.3% (95% CI: 6.8% to 34.8%) in women.

**Figure 3:**
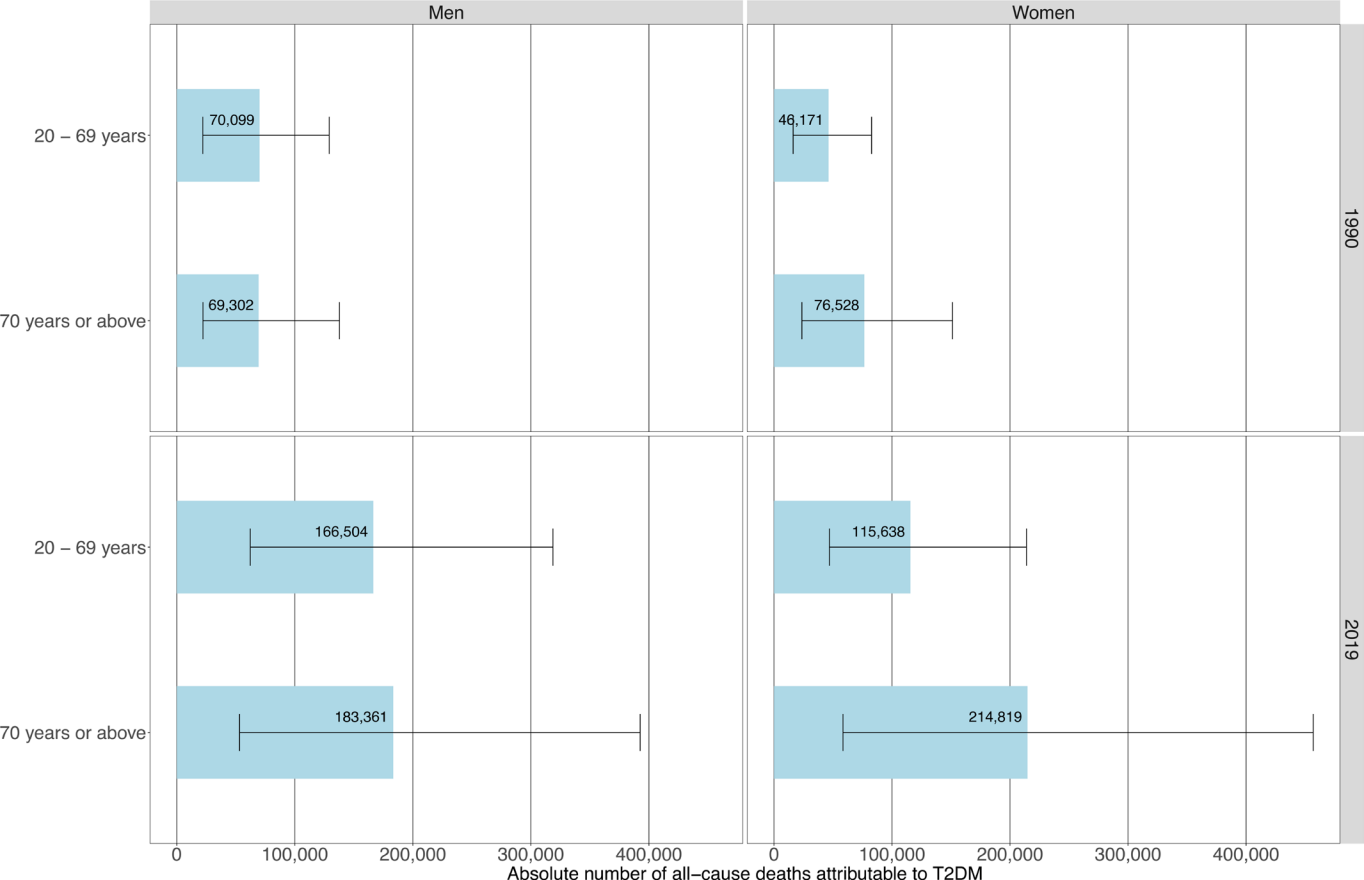
Absolute number of deaths attributable to type 2 diabetes mellitus (T2DM) in people aged <70 years and 70+ years by sex in 1990 and 2019.

In 2019, the absolute number of all-cause mortality attributable to 2014 T2DM prevalence in LAC is more than doubled the absolute number estimated in 1990 for both sexes (figure 3). Absolute number of all-cause deaths in 2019 attributable to 2014 T2DM prevalence were estimated at 349 787 (95% CI: 115 305 to 711 176) in men and 330 414 (95% CI: 105 399 to 670 664) in women.

### Results by country

At the country level, LAC countries with the largest proportions of all-cause mortality in 1990 attributable to 1985 T2DM prevalence in men were from the Caribbean and Southern Latin America; in women, these countries were mostly from the Caribbean (figure 1, online supplemental table 2). The largest proportions of all-cause deaths in 2019 attributable to 2014 T2DM prevalence were seen in the Caribbean for both sexes (figure 2, online supplemental table 2).

The countries with the largest proportions of all-cause deaths in 2019 attributable to 2014 T2DM prevalence in men were Bermuda (23.2% (95% CI: 8.0% to 40.6%)), Saint Kitts and Nevis (22.9% (95% CI: 9.8% to 37.0%)), and Saint Lucia (22.2% (95% CI: 7.4% to 39.2%)). On the other hand, the smallest proportions in men were in Peru (14.5% (95% CI: 4.4% to 27.7%)), Guatemala (14.6% (95% CI: 4.7% to 26.8%)), and Ecuador (14.6% (95% CI: 4.5% to 27.8%)); remarkably, two out of these three countries (Peru and Ecuador) are in Andean Latin America.

The largest proportions of all-cause deaths in 2019 attributable to 2014 T2DM prevalence in women were in Saint Kitts and Nevis (25.9% (95% CI: 10.3% to 42.9%)), Barbados (24.0% (95% CI: 8.4% to 42.2%)), and Saint Lucia (23.7% (95% CI: 7.2% to 43.0%)); conversely, the smallest proportions in women were seen in Haiti (16.3% (95% CI: 5.7% to 28.8%)), Peru (16.9% (95% CI: 5.3% to 31.9%)), and Paraguay (17.4% (95% CI: 5.9% to 32.2%)); these countries did not belong to the same subregion in LAC.

Over the study period, countries that consistently showed the largest absolute number of all-cause mortality attributable to T2DM were Brazil, Mexico, and Argentina (online supplemental table 2). In men, all-cause deaths in 2019 attributable to 2014 T2DM prevalence in these countries totaled 114 604 (95% CI: 41 392 to 214 053), 73 798 (95% CI: 23 969 to 154 623), and 33 170 (95% CI: 12 564 to 59 893), respectively. A similar profile was seen in women, where all-cause mortality in 2019 attributable to 2014 T2DM prevalence was estimated at 110 436 (95% CI: 38 042 to 207 758), 65 189 (95% CI: 21 496 to 134 257) and 31 430 (95% CI: 10 181 to 60 176), respectively.

The sensitivity analysis assuming a 10-year lag period between T2DM and all-cause mortality resulted in slightly smaller proportions of all-cause deaths attributable to T2DM compared with the main analysis assuming a 5-year lag period. The geographical patterns were identical to the main analysis (online supplemental figures 1,2). The sensitivity analysis restricted for diagnosed T2DM also revealed similar geographical patterns, but the proportions of all-cause deaths attributable to T2DM were consistently lower than in the main analysis (online supplemental figures 5,6).

### Results by age groups

In 1990, T2DM-attributable all deaths mostly occurred at old ages in women. Conversely, the absolute number of deaths at young versus old ages was similar in men (figure 3), though we observed a shift to older ages in deaths attributable to T2DM from 1990 to 2019 in both men and women. In 2019, T2DM-attributable deaths mostly occurred at older ages in both sexes; the proportions of deaths attributable to T2DM that occurred prematurely (below 70 years) were 47.6% in men and 35.0% in women (figure 3).

At the country level in 2019, Caribbean countries had the highest proportion of all-cause T2DM-attributable deaths occurring prematurely in both sexes. In men in 2019, countries with the highest proportions of all-cause deaths attributable to T2DM occurring prematurely were Saint Kitts and Nevis (66.4%), Guyana (65.2%), and Bahamas (61.3%); on the other hand, the lowest proportions in men were in Puerto Rico (37.6%), Uruguay (37.3%), and Cuba (35.4%).

In women in 2019, countries with the highest proportions of all-cause deaths occurring in people <70 years were in were Haiti (58.5%), Guyana (55.7%), and Belize (53.5%); conversely, the lowest proportions in women were in Puerto Rico (23.5%), Bermuda (22.7%), and Uruguay (20.7%).

### T2DM-attributable age-standardized all-cause mortality rates

**Figure 4:**
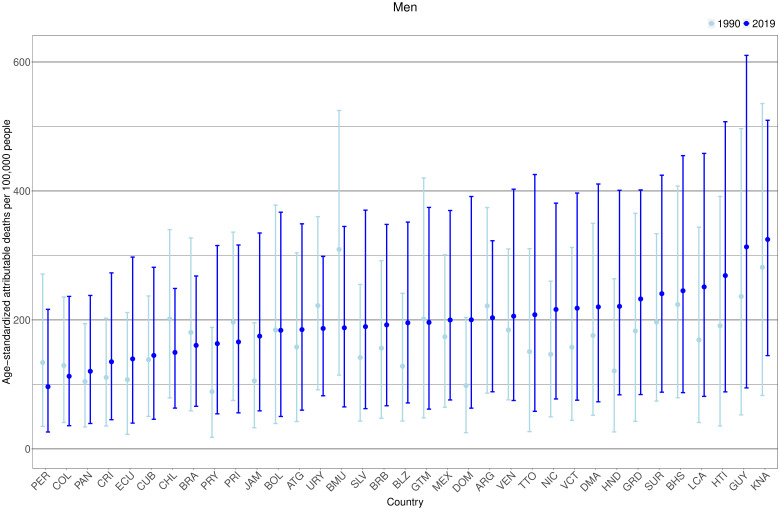
Age-standardized type 2 diabetes mellitus (T2DM)-attributable deaths per 100 000 people by country and year in men. Exact number estimates (along with their 95% CI) are presented in online supplemental table 2). Countries are presented in ascending order based on their death rates in 2019 (ie, countries with the highest rates in 2019 are on the right-hand side of the figure). *Countries are abbreviated according to the ISO3 code. From left to right, PER, Peru; COL, Colombia; PAN, Panama; CRI, Costa Rica; ECU, Ecuador; CUB, Cuba; CHL, Chile; BRA, Brazil; PRY, Paraguay; PRI, Puerto Rico; JAM, Jamaica; BOL, Bolivia; ATG, Antigua and Barbuda; URY, Uruguay; BMU, Bermuda; SLV, El Salvador; BRB, Barbados; BLZ, Belize; GTM, Guatemala; MEX, Mexico; DOM, Dominican Republic; ARG, Argentina; VEN, Venezuela; TTO, Trinidad and Tobago; NIC, Nicaragua; VCT, Saint Vincent and the Grenadines; DMA, Dominica; HND, Honduras; GRD, Grenada; SUR, Suriname; BHS, Bahamas; LCA, Saint Lucia; HTI, Haiti; GUY, Guyana; KNA, Saint Kitts and Nevis.

In men, we observed that 25 countries (out of 35) increased their T2DM-attributable age-standardized death rates from 1990 (figure 4, online supplemental table 2). In 2019 in men, the highest mortality rates were seen in Saint Kitts and Nevis (325 (95% CI: 145 to 510) deaths per 100 000), Guyana (313 (95% CI: 95 to 610) deaths per 100 000), and Haiti (269 (95% CI: 88 to 507) deaths per 100 000); all of them increased their mortality rates since 1990. On the other hand, the lowest mortality rates in 2019 for men were seen in Peru (96 (95% CI: 26 to 216) deaths per 100 000), Colombia (113 (95% CI: 36 to 236) deaths per 100 000), and Panama (120 (95% CI: 39 to 238) deaths per 100 000); the first two decreased their death rates since 1990.

**Figure 5:**
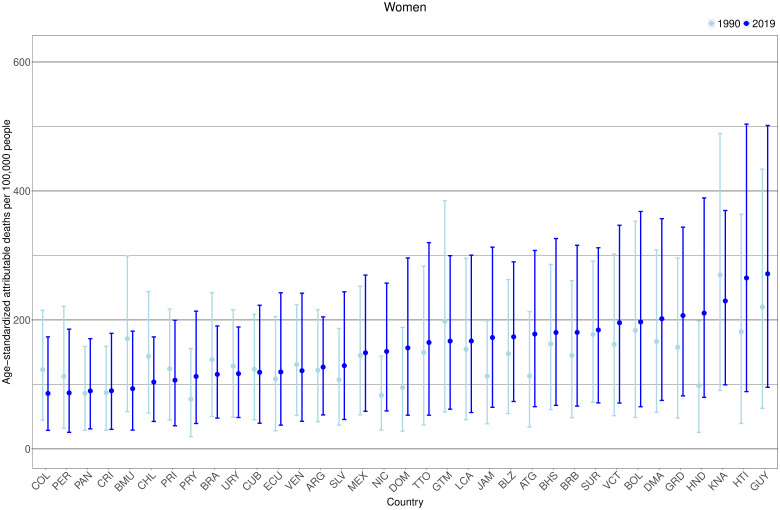
Age-standardized type 2 diabetes mellitus (T2DM)-attributable deaths per 100 000 people by country and year in women. Exact number estimates (along with their 95% CI) are presented in online supplemental table 2). Countries are presented in ascending order based on their death rates in 2019 (ie, countries with the highest rates in 2019 are on the right-hand side of the figure). *Countries are abbreviated according to the ISO3 code. From left to right, COL, Colombia; PER, Peru; PAN, Panama; CRI, Costa Rica; BMU, Bermuda; CHL, Chile; PRI, Puerto Rico; PRY, Paraguay; BRA, Brazil; URY, Uruguay; CUB, Cuba; ECU, Ecuador; VEN, Venezuela; ARG, Argentina; SLV, El Salvador; MEX, Mexico; NIC, Nicaragua; DOM, Dominican Republic; TTO, Trinidad and Tobago; GTM, Guatemala; LCA, Saint Lucia; JAM, Jamaica; BLZ, Belize; ATG, Antigua and Barbuda; BHS, Bahamas; BRB, Barbados; SUR, Suriname; VCT, Saint Vincent and the Grenadines; BOL, Bolivia; DMA, Dominica; GRD, Grenada; HND, Honduras; KNA, Saint Kitts and Nevis; HTI, Haiti; GUY, Guyana.

A similar pattern was observed in women, 24 countries (out of 35) increased their mortality rates from 1990 (figure 5, online supplemental table 2). In 2019 in women, countries with the highest mortality rates were Guyana (272 (95% CI: 96 to 502) deaths per 100 000), Haiti (265 (95% CI: 89 to 504) per 100 000), and Saint Kitts and Nevis (229 (95% CI: 99 to 370) deaths per 100 000); Guyana and Haiti increased their death rates since 1990. Notably, for both sexes, countries with the highest death rates were from the Caribbean. In women in 2019, countries with the lowest death rates were Colombia (86 (95% CI: 29 to 174) deaths per 100 000), Peru (87 (95% CI: 26 to 186) deaths per 100 000), and Panama (89 (95% CI: 31 to 171) deaths per 100 000); Colombia and Peru decreased their mortality rates since 1990.

The sensitivity analysis assuming a 10-year lag period between T2DM and all-cause mortality resulted in only slightly lower death rates compared with the main analysis (5-year lag period). Countries with the highest and lowest death rates were the same in both analyses (online supplemental figures 3,4). The sensitivity analysis restricted to diagnosed diabetes revealed lower attributable death rates (online supplemental figures 7,8) than the main analysis for total T2DM; nonetheless, the countries with the highest and lowest rates were also consistent in both analyses.

### Correlations between the T2DM-attributable death rates and economic metrics

For the 2019 age-standardized T2DM-attributable death rates at the country level, there was a negative correlation with the 2019 HDI, yet a positive correlation with the proportion of people living in rural areas in 2019 (online supplemental figure 9). Of note, there was a negative correlation between the 1990–2019 variation in age-standardized death rates and the 1990–2019 variation in GDP per capita.

## Discussion

We estimated all-cause mortality attributable to T2DM in 35 countries in LAC using prevalence and mortality data from global estimates,^1 3^ and RRs from a meta-analysis of cohort studies in LAC.^18^ We observed an increase in the proportion and the absolute number of all-cause deaths attributable to T2DM in the last 30 years in LAC; although the increase in the proportion of all-cause deaths attributable to T2DM was similar in both sexes, the proportions were always higher in women compared with men. There was also a shift to older ages in the number of attributable deaths to T2DM in both sexes; in 2019, almost one out of two deaths in men occurred in those aged <70 years, whereas one out of three deaths in women occurred in those aged <70 years. Notably, Caribbean countries showed the highest proportions of all-cause deaths attributable to T2DM, highest proportions of deaths occurring prematurely, and highest T2DM age-standardized death rates. The countries where GDP per capita increased the most from 1990 to 2019 reduced their death rates throughout the same period. There was a positive correlation of the death rates with rurality, yet a negative correlation with HDI.

### Public health relevance

Our results suggest that countries with the highest burden (ie, proportions and death rates) attributable to T2DM were from the Caribbean. This finding calls for urgent actions in these countries. For example, they could aim to reduce the mortality burden associated with T2DM, especially in people aged <70 years. We encourage LAC countries to improve rates of diagnosis of T2DM, which can be done by validating T2DM screening tools (eg, Finnish Diabetes Risk Score)^29^ for LAC populations. Also, as LAC public health systems often face limited resources, low-cost interventions to improve T2DM management and prevention of complications are warranted.^30^ For example, interventions using mobile health technology (eg, telemedicine) and community health workers could be used;^31 32^ remarkably, some efforts in LAC have been implemented and more are currently being evaluated.^33–38^ As these interventions could help to accomplish NCDs mortality reduction targets,^19 20^ the findings herein presented could serve as a baseline to inform the surveillance and monitoring of LAC countries as they work to achieve these international goals.

### Potential explanations

As the PAF computation leveraged on country-specific prevalence estimates and constant RRs for all countries and both sexes, the proportion of all-cause deaths attributable to T2DM was largely driven by the country-specific T2DM prevalence estimates. We observed that Caribbean countries had the highest proportion of all-cause deaths in 2019 attributable to 2014 T2DM prevalence. This is consistent with the fact that Caribbean countries had the highest T2DM prevalence in 2014 across LAC.^3^ We found that the proportion of all-cause deaths attributable to T2DM was slightly higher in women compared with men in LAC. This is consistent with sex-specific T2DM prevalence estimates in LAC, where the prevalence of T2DM is higher in women than men.^3^ The factors behind the rise in T2DM prevalence could also explain the upward trend of the proportion of all-cause deaths attributable to T2DM in LAC. For example, high body mass index is the most important risk factor for T2DM in adults and obesity prevalence in LAC has more than quadrupled in men and almost tripled in women from 1975 to 2016.^39^

Our sensitivity analysis restricted to diagnosed T2DM resulted in lower proportion of all-cause deaths attributable to T2DM compared with our main analysis for total T2DM (undiagnosed and diagnosed). This is explained by the high proportion of people with T2DM that are unaware of their diagnosis in LAC.^2 28^ Of note, we only used one estimate of the proportion of diagnosed diabetes in LAC. Therefore, these sensitivity results for LAC countries where access to healthcare is much more limited could have been underestimated; conversely, these sensitivity results for countries where access to healthcare is optimal could have been overestimated.

The top three countries with the absolute number of all-cause deaths attributable to T2DM were Brazil, Mexico, and Argentina; these three countries accounted for 63% of the total absolute number of all-cause deaths in 2019 attributable to 2014 T2DM prevalence in LAC. An explanation for these results could be the large population of these countries, as 61% of all adults ≥20 years in LAC live in Brazil, Mexico, and Argentina.^21^ Furthermore, Brazil and Mexico were positioned in the top 10 countries with the largest number of adults with T2DM in 2014.^3^

### Results in context

The study most similar to ours (ie, same methodology, exposure, and outcome) provided global, regional, and national results of all-cause deaths attributable to diabetes.^40^ Nonetheless, they only provided estimates for people aged 20–79 years, only used RR derived from US populations for LAC, and only analyzed for 1 year.^40^ Our results advanced this evidence by incorporating RR from LAC in the analysis, providing—arguably—more accurate results for LAC.

Compared with their proportion and absolute estimates of all-cause deaths attributable to T2DM for South and Central America in 2019,^40^ our estimates for the same year were higher. This could be explained by two factors. First, we included a broader population (ie, all people ≥20 years). Second, our RRs were higher compared with theirs in the older age groups. Nevertheless, theirs and our results agreed on the observation that most deaths attributable to T2DM occurred in older age groups.

Another comparative risk assessment similar to ours was developed by the GBD.^16 21^ Even though they analyzed different exposures (high fasting plasma glucose), we both found that the highest death rates were seen in Caribbean countries; in women, theirs and our findings signaled the same countries with the highest death rates (Haiti and Guyana). Also, similar to our results, they reported a shift to older ages (≥70 years) in the absolute number of attributable deaths between 1990 and 2019.

National efforts have been made in LAC countries to describe the mortality attributable to T2DM based on death certificates.^4 6 7^ In this approach, deaths would be considered as caused by T2DM if they had it listed as their UCD. Although this is the usual approach when analyzing causes of death, it has limitations to consider. First, T2DM is not usually certified as the UCD in VR of patients with T2DM (eg, even less than 15%).^11 12^ Second, LAC countries may not meet high levels of registered deaths; also, these registries may have quality issues (eg, garbage codes). Both factors could explain the differences between the results we presented following an estimation approach and the results of studies using death certificates.^4 6 41^ For instance, we reported a higher proportion of all deaths attributable to T2DM, a higher absolute number of deaths attributable to T2DM, and a higher T2DM-attributable age-standardized death rates, than the ones reported in Argentina,^6^ Brazil,^41^ Chile,^6^ Colombia,^6^ Mexico^6^ and Peru.^4^

### Strengths and limitations

In this study, we used nationally representative data sources from 35 countries in LAC, along with RR derived from a recently published meta-analysis of cohort studies conducted in LAC.^18^ Furthermore, we leveraged on prevalence estimates (NCD-RisC) that accounted for temporal changes in T2DM diagnostic criteria and included both diagnosed and undiagnosed T2DM.^3^ To our knowledge, this is the first effort to estimate the T2DM-attributable mortality burden in LAC using RR derived from cohorts in LAC and considered a lag period between exposure (T2DM diagnosis) and outcome (all-cause mortality). Nonetheless, there are some limitations we must acknowledge. First, although the RRs of mortality were retrieved from a meta-analysis of cohorts in LAC,^18^ these may not represent the mortality risk profile from all LAC countries because they did not pool risk estimates from all countries in LAC. The RR of all-cause mortality in people with versus without T2DM is unlikely to be equal among all LAC countries^18 40^ because there are heterogeneous profiles in terms of healthcare access and T2DM-related policies.^30^ Second, we assumed the same RR for men and women because of data availability. Even though the meta-analysis reported similar (non-age-specific) RR for men and women,^18^ international evidence describes that RR differs by sex.^12 42^ Future research in LAC is needed to produce more robust data on the mortality attributable to T2DM by sex and including other causes of death. For instance, cohort studies in LAC should aim to analyze cause-specific mortality in people with T2DM versus people without T2DM, and differences in the all-cause mortality by sex, urban/rural location, and socioeconomic status. Third, because of data availability, age-specific RRs were derived from LAC cohorts based on self-reported diabetes. As local evidence indicates the RR of all-cause mortality in people with self-reported diabetes is slightly higher than in people with total diabetes,^18^ our main estimates could have been overestimated because we used RR for self-reported diabetes only. We performed a sensitivity analysis restricted for diagnosed diabetes only which resulted in smaller metrics than those from the main analysis, though the main findings and conclusions did not change (eg, ranking of countries). Readers and potential users of this information should interpret our findings in light of this limitation. Finally, prevalence estimates for 11 Caribbean countries (online supplemental table 2) included in our analysis were modeled estimates. This could have explained the higher uncertainty for these countries compared with those countries which prevalence estimates were based on local data (eg, Brazil).

## Conclusion

From 1990 to 2019, there was an increase in the proportion and the absolute number of all-cause mortality attributable to T2DM across LAC. Furthermore, most LAC countries increased their T2DM-attributable death rates since 1990. These findings call to strengthen early diagnosis and management of T2DM, along with prevention of T2DM microvascular and cardiovascular complications.

## Data Availability

All data relevant to the study are included in the article or uploaded as supplementary information. Datasets and analysis code in supplementary materials.
