# Supplementary material for: Mortality attributable to type 2 diabetes mellitus in Latin America and the Caribbean: a comparative risk assessment analysis
Source: BMJ Open Diabetes Res Care. 2022 Feb 19;10(1):e002673. doi: 10.1136/bmjdrc-2021-002673 (PMC8860056; doi:10.1136/bmjdrc-2021-002673)

SUPPLEMENTARY MATERIALS

Mortality attributable to type 2 diabetes mellitus in Latin America and the Caribbean: a comparative risk assessment analysis

Correspondence author:

Rodrigo M Carrillo-Larco, MD

Department of Epidemiology and Biostatistics

School of Public Health

Imperial College London

rcarrill@ic.ac.uk

Guzman-Vilca WC, Carrillo-Larco RM. *BMJ Open Diab Res Care* 2022; 10:e002673. doi: 10.1136/bmjdrc-2021-002673

Table of Contents

Supplementary table 1: Relative risks (RRs) of all-cause mortality in population with vs without type 2 diabetes mellitus (T2DM) 3

Supplementary table 2: population attributable fractions (PAF), absolute number of all-cause deaths, and age-standardised death rates attributable to T2DM by country and sex in 1990 and 2019 4

Supplementary figure 2: Sensitivity analysis assuming a 10-year lag period | Percentage of deaths in 2019 attributable to 2009 type 2 diabetes mellitus (T2DM) prevalence by country and sex 13

Supplementary figure 3: Sensitivity analysis assuming a 10-year lag period | Age-standardised type 2 diabetes mellitus (T2DM) attributable deaths per 100,000 people by country and year in men 14

Supplementary figure 4: Sensitivity analysis assuming a 10-year lag period | Age-standardised type 2 diabetes mellitus (T2DM) attributable deaths per 100,000 people by country and year in women 15

Supplementary figure 5: Sensitivity analysis restricted for diagnosed diabetes only | Percentage of deaths in 1990 attributable to 1985 type 2 diabetes mellitus (T2DM) prevalence by country and sex 16

Supplementary figure 6: Sensitivity analysis restricted for diagnosed diabetes only | Percentage of deaths in 2019 attributable to 2014 type 2 diabetes mellitus (T2DM) prevalence by country and sex 17

Supplementary figure 7: Sensitivity analysis restricted for diagnosed diabetes only | Age-standardised type 2 diabetes mellitus (T2DM) attributable deaths per 100,000 people by country and year in men 18

Supplementary figure 8: Sensitivity analysis restricted for diagnosed diabetes only | Age-standardised type 2 diabetes mellitus (T2DM) attributable deaths per 100,000 people by country and year in women 19

Supplementary figure 9: Correlations between the age-standardised T2DM-attributable death rates and economic metrics 20

Supplementary table 1: Relative risks (RRs) of all-cause mortality in population with vs without type 2 diabetes mellitus (T2DM)

Age group	RR for all-cause mortality (95% IC)
**20 to 24**	2.80 (1.46-5.39)
**25 to 29**	2.74 (1.55-4.85)
**30 to 34**	2.69 (1.65-4.37)
**35 to 39**	2.63 (1.75-3.95)
**40 to 44**	2.58 (1.86-3.59)
**45 to 49**	2.53 (1.95-3.28)
**50 to 54**	2.48 (2.01-3.05)
**55 to 59**	2.43 (2.01-2.92)
**60 to 64**	2.38 (1.94-2.91)
**65 to 69**	2.33 (1.81-3.00)
**70 to 74**	2.28 (1.66-3.14)
**75 to 79**	2.23 (1.50-3.32)
**80 to 84**	2.19 (1.36-3.53)
**85 plus**	2.13 (1.19-3.81)

These RRs were derived from a meta-analysis of cohorts conducted in LAC population.

Reference: Carrillo-Larco RM, Barengo NC, Albitres-Flores L, Bernabe-Ortiz A. The risk of mortality among people with type 2 diabetes in Latin America: A systematic review and meta-analysis of population-based cohort studies. Diabetes Metab Res Rev. 2019;35(4):e3139.

Supplementary table 2: population attributable fractions (PAF), absolute number of all-cause deaths, and age-standardised death rates attributable to T2DM by country and sex in 1990 and 2019

Country	Year (for deaths)	Sex	PAF	PAF lower limit (95% CI)	PAF upper limit (95% CI)	Attributable deaths	Attributable deaths lower limit (95% CI)	Attributable deaths upper limit (95% CI)	Death rate per 100,000 people	Death rate per 100,000 people lower limit (95% CI)	Death rate per 100,000 people upper limit (95% CI)
Argentina	1990	Men	0.17	0.06	0.29	20914	7833	37181	222	86	374
Argentina	1990	Women	0.16	0.05	0.3	15941	5108	30358	122	42	216
Chile	1990	Men	0.15	0.06	0.28	5897	2161	10677	201	79	340
Chile	1990	Women	0.18	0.06	0.32	5302	1902	9719	144	55	244
Uruguay	1990	Men	0.17	0.07	0.3	2585	980	4607	222	92	360
Uruguay	1990	Women	0.17	0.06	0.32	2197	731	4180	129	49	216
Antigua and Barbuda*	1990	Men	0.12	0.03	0.23	25	7	52	158	42	304
Antigua and Barbuda	1990	Women	0.16	0.04	0.3	30	8	62	113	34	213
Bahamas*	1990	Men	0.13	0.05	0.24	96	32	184	224	79	408
Bahamas	1990	Women	0.17	0.06	0.3	95	32	181	163	61	286
Barbados	1990	Men	0.14	0.04	0.26	140	41	281	156	47	292
Barbados	1990	Women	0.18	0.06	0.34	203	61	403	145	48	261
Belize	1990	Men	0.12	0.04	0.23	40	12	82	128	43	241
Belize	1990	Women	0.17	0.06	0.31	50	16	97	148	55	263
Cuba	1990	Men	0.13	0.04	0.26	4969	1569	9590	138	50	237

**Cuba**	1990	Women	0.16	0.05	0.3	4681	1476	8911	124	45	209
**Dominica**	1990	Men	0.12	0.04	0.24	37	11	79	176	52	350
**Dominica**	1990	Women	0.17	0.06	0.32	56	17	114	167	57	309
**Dominican Republic**	1990	Men	0.09	0.02	0.19	1161	274	2606	98	25	204
**Dominican Republic**	1990	Women	0.12	0.04	0.24	1136	301	2439	95	27	188
**Grenada***	1990	Men	0.11	0.02	0.22	41	9	90	183	43	365
**Grenada**	1990	Women	0.15	0.04	0.29	57	15	118	158	48	296
**Guyana***	1990	Men	0.09	0.02	0.18	268	54	600	236	53	497
**Guyana**	1990	Women	0.13	0.04	0.24	275	72	581	220	63	434
**Haiti***	1990	Men	0.08	0.01	0.16	1703	278	3857	191	35	392
**Haiti**	1990	Women	0.08	0.02	0.16	1816	347	4059	182	39	364
**Jamaica**	1990	Men	0.12	0.03	0.23	628	180	1257	106	33	196
**Jamaica**	1990	Women	0.15	0.05	0.29	841	255	1632	113	39	199
**Saint Lucia***	1990	Men	0.1	0.03	0.21	41	10	87	169	41	344
**Saint Lucia**	1990	Women	0.14	0.04	0.28	55	15	112	154	45	296
**Saint Vincent and the Grenadines***	1990	Men	0.11	0.03	0.22	33	9	70	158	44	312
**Saint Vincent and the Grenadines**	1990	Women	0.15	0.05	0.29	48	14	96	162	51	302
**Suriname***	1990	Men	0.14	0.05	0.25	159	52	301	197	74	334
**Suriname**	1990	Women	0.17	0.06	0.32	169	56	317	177	72	291

Guzman-Vilca WC, Carrillo-Larco RM. *BMJ Open Diab Res Care* 2022; 10:e002673. doi: 10.1136/bmjdrc-2021-002673

**Trinidad and Tobago***	1990	Men	0.09	0.02	0.2	381	69	821	151	27	311
**Trinidad and Tobago**	1990	Women	0.14	0.03	0.26	457	109	925	150	37	284
**Bolivia**	1990	Men	0.1	0.02	0.21	1670	332	3646	184	39	378
**Bolivia**	1990	Women	0.12	0.03	0.23	1974	481	4087	184	49	353
**Ecuador**	1990	Men	0.09	0.02	0.2	1855	349	3929	107	23	211
**Ecuador**	1990	Women	0.12	0.03	0.24	1915	457	3882	108	28	205
**Peru**	1990	Men	0.11	0.03	0.22	5133	1147	11395	134	35	271
**Peru**	1990	Women	0.13	0.04	0.25	4674	1162	10110	113	32	221
**Colombia**	1990	Men	0.09	0.03	0.18	7025	1995	13944	129	41	236
**Colombia**	1990	Women	0.14	0.05	0.26	7059	2308	13441	123	45	215
**Costa Rica**	1990	Men	0.12	0.03	0.23	634	177	1295	111	36	203
**Costa Rica**	1990	Women	0.13	0.04	0.26	549	159	1123	87	29	159
**El Salvador**	1990	Men	0.1	0.03	0.19	1318	357	2602	142	43	255
**El Salvador**	1990	Women	0.14	0.04	0.26	1189	357	2283	107	37	187
**Guatemala**	1990	Men	0.09	0.02	0.19	2220	466	5048	201	48	420
**Guatemala**	1990	Women	0.12	0.04	0.22	2032	544	4285	198	57	385
**Honduras**	1990	Men	0.09	0.02	0.18	786	152	1863	121	26	264
**Honduras**	1990	Women	0.1	0.03	0.19	669	155	1484	98	26	199
**Mexico**	1990	Men	0.13	0.05	0.25	23421	7958	43532	174	65	301
**Mexico**	1990	Women	0.16	0.05	0.29	20468	7009	37906	145	53	252
**Nicaragua**	1990	Men	0.12	0.04	0.23	624	195	1200	147	50	260

Guzman-Vilca WC, Carrillo-Larco RM. *BMJ Open Diab Res Care* 2022; 10:e002673. doi: 10.1136/bmjdrc-2021-002673

**Nicaragua**	1990	Women	0.13	0.04	0.25	459	137	888	83	29	144
**Panama**	1990	Men	0.11	0.03	0.22	526	156	1050	105	34	194
**Panama**	1990	Women	0.14	0.04	0.26	448	133	896	86	29	159
**Venezuela**	1990	Men	0.14	0.05	0.26	5429	2054	9835	184	76	310
**Venezuela**	1990	Women	0.15	0.06	0.28	4432	1596	8221	131	52	224
**Brazil**	1990	Men	0.11	0.03	0.21	46684	14326	89476	181	59	327
**Brazil**	1990	Women	0.14	0.05	0.26	40926	14111	76077	138	50	242
**Paraguay**	1990	Men	0.1	0.02	0.2	600	108	1382	89	18	188
**Paraguay**	1990	Women	0.11	0.03	0.23	632	139	1398	77	19	156
**Bermuda***	1990	Men	0.2	0.07	0.35	55	19	99	309	114	525
**Bermuda**	1990	Women	0.21	0.07	0.38	44	13	83	171	58	299
**Puerto Rico**	1990	Men	0.16	0.06	0.29	2274	807	4184	196	75	336
**Puerto Rico**	1990	Women	0.18	0.06	0.33	1789	581	3385	124	45	217
**Saint Kitts and Nevis***	1990	Men	0.15	0.05	0.27	32	10	63	282	83	536
**Saint Kitts and Nevis**	1990	Women	0.18	0.06	0.32	41	14	80	270	91	489
**Argentina**	2019	Men	0.19	0.07	0.34	33170	12564	59893	203	89	323
**Argentina**	2019	Women	0.19	0.06	0.36	31430	10181	60176	127	53	205
**Chile**	2019	Men	0.19	0.08	0.34	11303	4330	20410	150	63	249
**Chile**	2019	Women	0.21	0.07	0.38	10774	3703	20197	104	43	174
**Uruguay**	2019	Men	0.18	0.07	0.33	3040	1127	5620	187	82	298
**Uruguay**	2019	Women	0.2	0.06	0.38	3279	984	6436	117	49	189

**Antigua and Barbuda***	2019	Men	0.19	0.06	0.34	57	17	118	185	60	349
**Antigua and Barbuda**	2019	Women	0.23	0.08	0.41	66	21	129	178	66	308
**Bahamas***	2019	Men	0.19	0.07	0.33	284	90	589	245	87	455
**Bahamas**	2019	Women	0.23	0.09	0.39	261	84	534	181	68	326
**Barbados**	2019	Men	0.2	0.07	0.36	301	92	613	192	67	348
**Barbados**	2019	Women	0.24	0.08	0.42	371	113	741	181	66	316
**Belize**	2019	Men	0.16	0.06	0.3	183	57	372	196	71	352
**Belize**	2019	Women	0.23	0.09	0.4	161	57	306	174	74	290
**Cuba**	2019	Men	0.16	0.05	0.31	9393	2535	20817	145	46	282
**Cuba**	2019	Women	0.2	0.06	0.37	9558	2595	20708	119	40	223
**Dominica**	2019	Men	0.18	0.06	0.31	67	19	140	220	73	411
**Dominica**	2019	Women	0.23	0.08	0.4	73	22	150	202	75	357
**Dominican Republic**	2019	Men	0.16	0.06	0.29	6020	1643	13378	200	63	391
**Dominican Republic**	2019	Women	0.2	0.07	0.36	5342	1506	11549	157	52	296
**Grenada***	2019	Men	0.18	0.07	0.31	76	26	142	233	84	402
**Grenada**	2019	Women	0.23	0.08	0.4	85	29	159	207	82	344
**Guyana***	2019	Men	0.17	0.06	0.29	591	158	1276	313	95	610
**Guyana**	2019	Women	0.22	0.09	0.37	589	185	1207	272	96	502
**Haiti***	2019	Men	0.16	0.06	0.28	5545	1532	12490	269	88	507
**Haiti**	2019	Women	0.16	0.06	0.29	6091	1711	13787	265	89	504

**Jamaica**	2019	Men	0.18	0.06	0.32	1747	500	3776	175	59	335
**Jamaica**	2019	Women	0.23	0.08	0.4	2083	587	4392	173	65	313
**Saint Lucia***	2019	Men	0.22	0.07	0.39	171	49	345	251	81	458
**Saint Lucia**	2019	Women	0.24	0.07	0.43	141	38	291	167	56	300
**Saint Vincent and the Grenadines***	2019	Men	0.18	0.06	0.33	101	32	203	218	76	397
**Saint Vincent and the Grenadines**	2019	Women	0.21	0.07	0.38	92	29	182	196	71	347
**Suriname***	2019	Men	0.2	0.08	0.34	441	144	870	241	88	424
**Suriname**	2019	Women	0.23	0.09	0.39	417	138	803	184	71	312
**Trinidad and Tobago***	2019	Men	0.19	0.06	0.34	1236	317	2791	208	58	426
**Trinidad and Tobago**	2019	Women	0.23	0.08	0.39	1145	312	2486	165	52	320
**Bolivia**	2019	Men	0.15	0.05	0.28	4938	1225	10940	184	50	367
**Bolivia**	2019	Women	0.19	0.06	0.33	5952	1782	12385	197	65	368
**Ecuador**	2019	Men	0.15	0.05	0.28	6787	1705	16252	140	40	297
**Ecuador**	2019	Women	0.18	0.06	0.33	6664	1754	15312	119	37	242
**Peru**	2019	Men	0.14	0.04	0.28	10669	2523	26275	96	26	216
**Peru**	2019	Women	0.17	0.05	0.32	10999	2762	26030	87	26	186
**Colombia**	2019	Men	0.16	0.05	0.29	19365	5442	44643	113	36	236
**Colombia**	2019	Women	0.18	0.06	0.33	19346	5482	43428	86	29	174
**Costa Rica**	2019	Men	0.17	0.06	0.31	2240	642	5111	135	45	273

**Costa Rica**	2019	Women	0.18	0.06	0.35	1910	520	4359	90	30	179
**El Salvador**	2019	Men	0.16	0.05	0.29	3381	901	7630	190	62	370
**El Salvador**	2019	Women	0.2	0.07	0.36	3398	917	7494	129	46	244
**Guatemala**	2019	Men	0.15	0.05	0.27	6624	1720	15016	196	62	374
**Guatemala**	2019	Women	0.19	0.07	0.33	6953	2055	14834	167	62	300
**Honduras**	2019	Men	0.17	0.07	0.29	4071	1401	8167	221	84	401
**Honduras**	2019	Women	0.19	0.08	0.32	4348	1506	8885	211	80	389
**Mexico**	2019	Men	0.19	0.07	0.33	73798	23969	154623	200	76	370
**Mexico**	2019	Women	0.21	0.08	0.37	65189	21496	134257	149	58	270
**Nicaragua**	2019	Men	0.18	0.07	0.31	2688	834	5488	216	77	381
**Nicaragua**	2019	Women	0.21	0.08	0.35	2384	790	4723	151	59	257
**Panama**	2019	Men	0.17	0.06	0.3	1706	472	3836	120	39	238
**Panama**	2019	Women	0.19	0.06	0.34	1483	409	3257	90	31	171
**Venezuela**	2019	Men	0.18	0.08	0.32	18596	6022	40625	206	75	403
**Venezuela**	2019	Women	0.18	0.07	0.33	13430	4028	30427	121	43	241
**Brazil**	2019	Men	0.16	0.06	0.28	114604	41392	214053	161	66	268
**Brazil**	2019	Women	0.19	0.07	0.34	110436	38042	207758	116	48	191
**Paraguay**	2019	Men	0.16	0.05	0.29	2859	776	6485	163	54	315
**Paraguay**	2019	Women	0.17	0.06	0.32	2355	640	5350	112	39	214
**Bermuda***	2019	Men	0.23	0.08	0.41	76	23	154	188	65	345
**Bermuda**	2019	Women	0.23	0.06	0.44	57	13	128	93	29	183
**Puerto Rico**	2019	Men	0.22	0.08	0.38	3670	1021	8071	166	56	316
**Puerto Rico**	2019	Women	0.23	0.07	0.42	3559	906	7835	107	36	200

**Saint Kitts and Nevis***	2019	Men	0.23	0.1	0.37	65	25	118	325	145	510
**Saint Kitts and Nevis**	2019	Women	0.26	0.1	0.43	51	18	98	229	99	370

*T2DM prevalence estimates for Antigua and Barbuda, Bahamas, Bermuda, Grenada, Guyana, Haiti, Saint Kitts and Nevis, Saint Lucia, Saint Vincent and the Grenadines, Suriname and Trinidad and Tobago were modelled estimates.

Reference: NCD Risk Factor Collaboration (NCD-RisC). Worldwide trends in diabetes since 1980: a pooled analysis of 751 population-based studies with 4.4 million participants. Lancet. 2016 09;387(10027):1513–30.

Supplementary figure 1: Sensitivity analysis assuming a 10-year lag period | Percentage of deaths in 1990 attributable to 1980 type 2 diabetes mellitus (T2DM) prevalence by country and sex

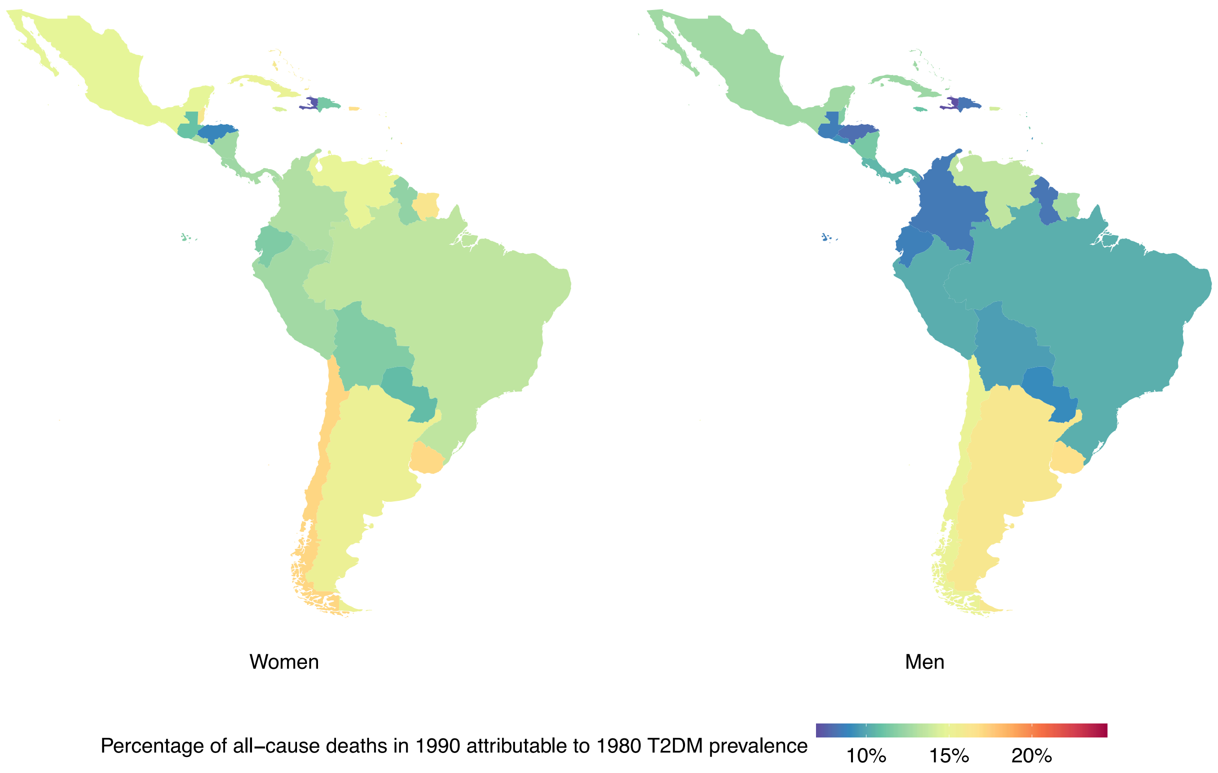

Guzman-Vilca WC, Carrillo-Larco RM. *BMJ Open Diab Res Care* 2022; 10:e002673. doi: 10.1136/bmjdrc-2021-002673

Supplementary figure 2: Sensitivity analysis assuming a 10-year lag period | Percentage of deaths in 2019 attributable to 2009 type 2 diabetes mellitus (T2DM) prevalence by country and sex

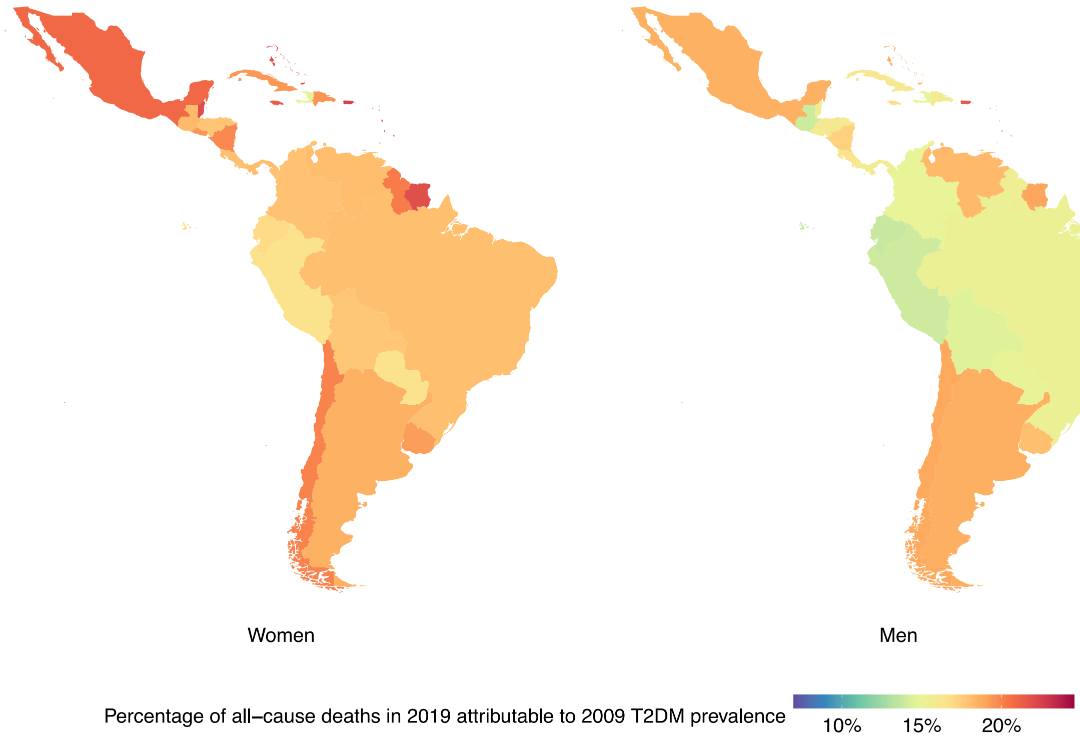

Supplementary figure 3: Sensitivity analysis assuming a 10-year lag period | Age-standardised type 2 diabetes mellitus (T2DM) attributable deaths per 100,000 people by country and year in men

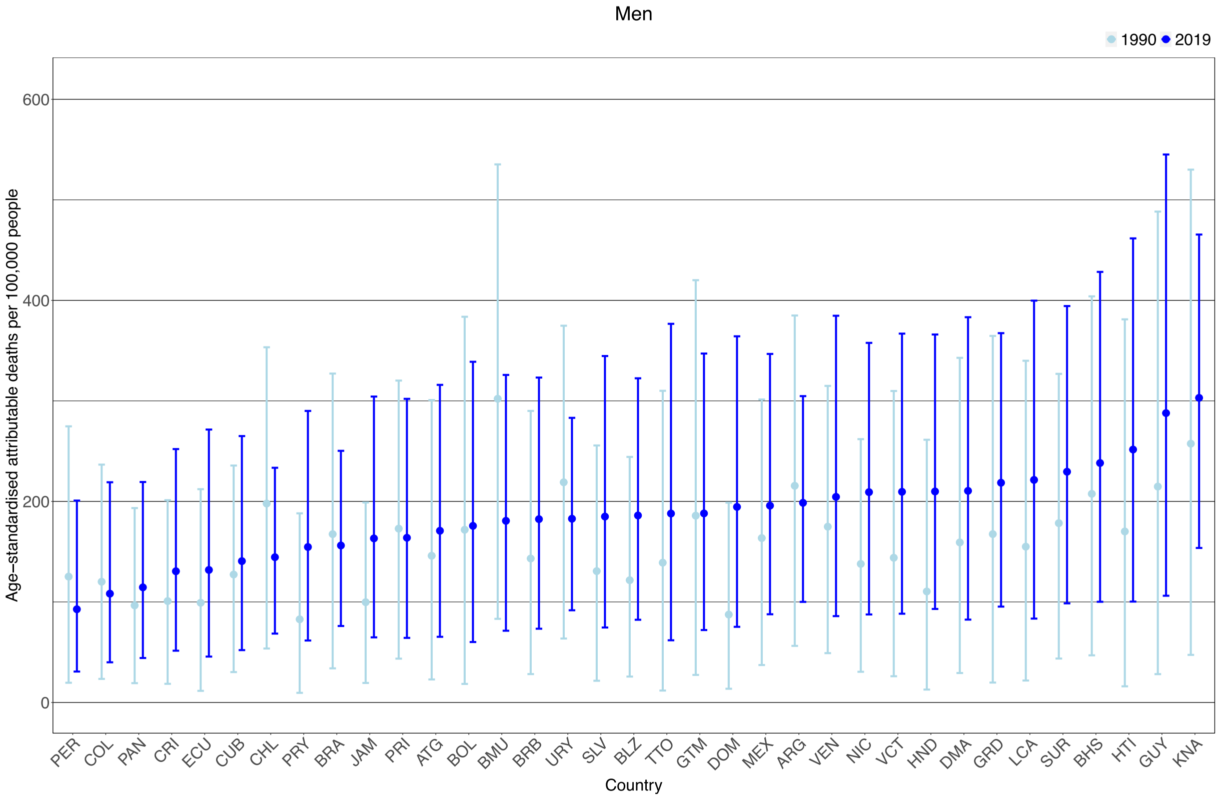

Guzman-Vilca WC, Carrillo-Larco RM. *BMJ Open Diab Res Care* 2022; 10:e002673. doi: 10.1136/bmjdrc-2021-002673

Supplementary figure 4: Sensitivity analysis assuming a 10-year lag period | Age-standardised type 2 diabetes mellitus (T2DM) attributable deaths per 100,000 people by country and year in women

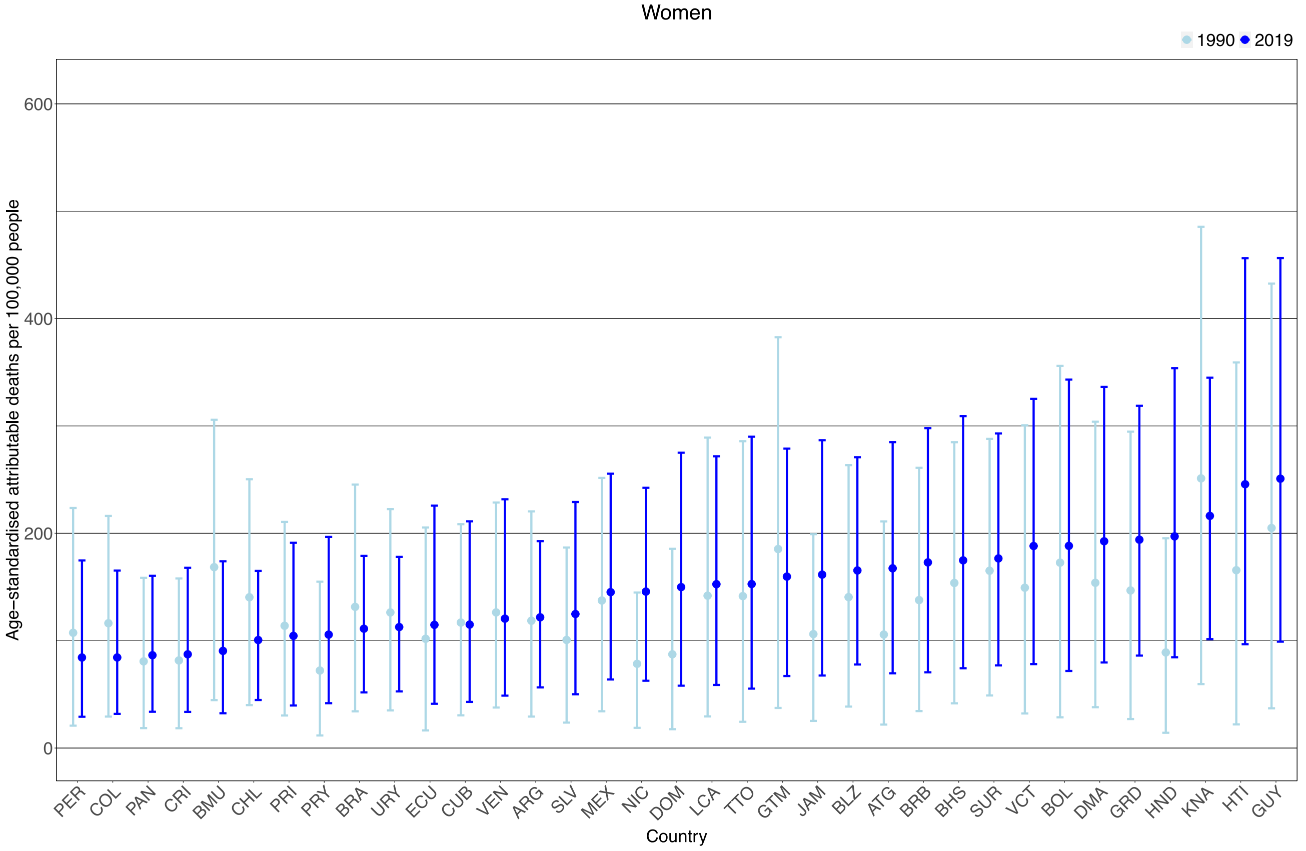

Guzman-Vilca WC, Carrillo-Larco RM. *BMJ Open Diab Res Care* 2022; 10:e002673. doi: 10.1136/bmjdrc-2021-002673

Supplementary figure 5: Sensitivity analysis restricted for diagnosed diabetes only | Percentage of deaths in 1990 attributable to 1985 type 2 diabetes mellitus (T2DM) prevalence by country and sex

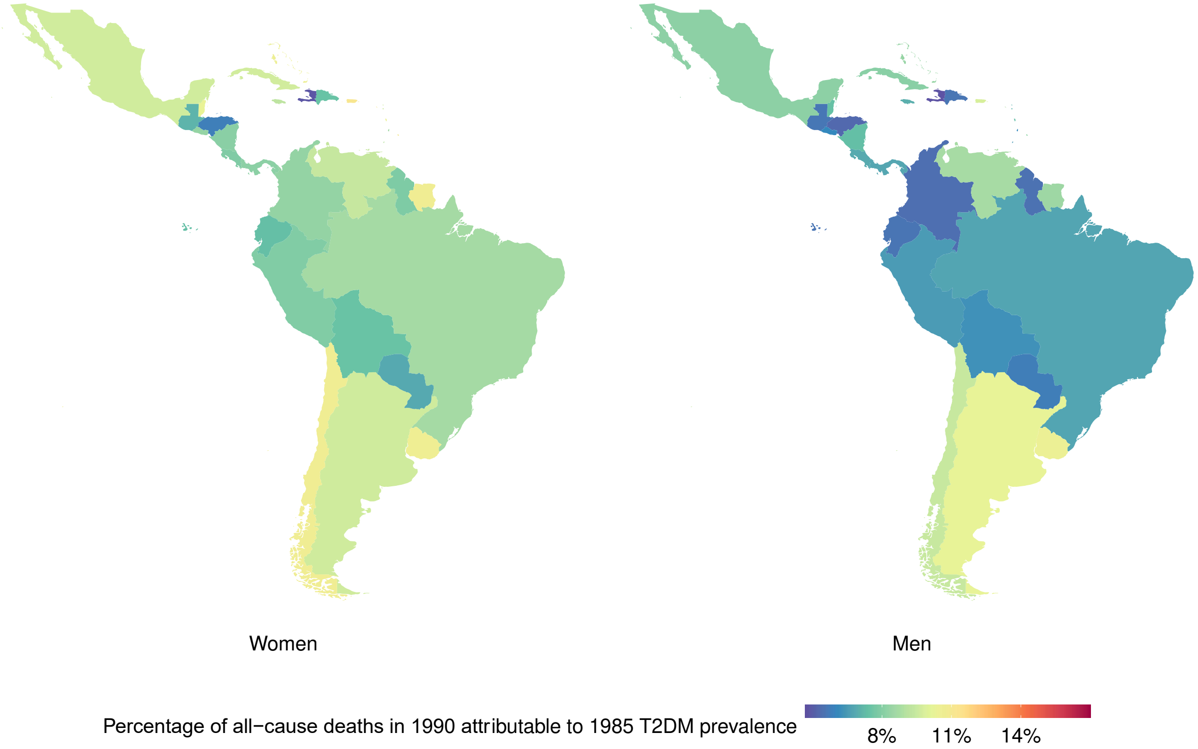

Supplementary figure 6: Sensitivity analysis restricted for diagnosed diabetes only | Percentage of deaths in 2019 attributable to 2014 type 2 diabetes mellitus (T2DM) prevalence by country and sex

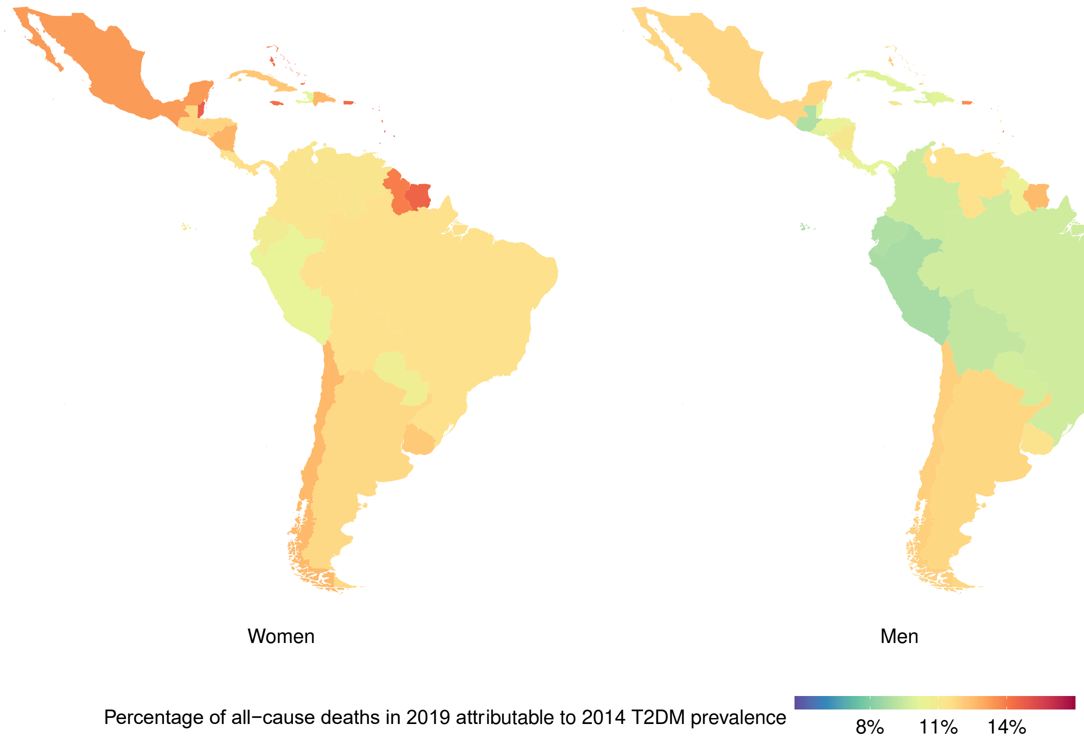

Supplementary figure 7: Sensitivity analysis restricted for diagnosed diabetes only | Age-standardised type 2 diabetes mellitus (T2DM) attributable deaths per 100,000 people by country and year in men

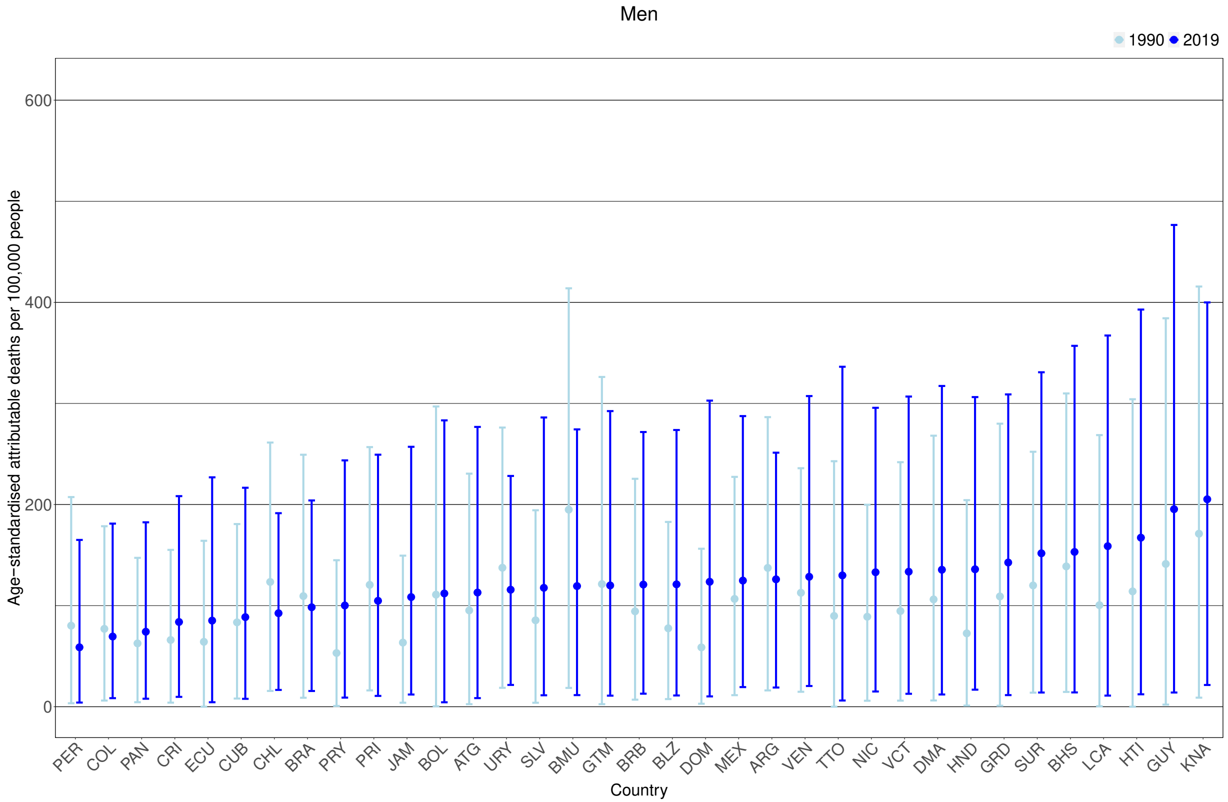

Supplementary figure 8: Sensitivity analysis restricted for diagnosed diabetes only | Age-standardised type 2 diabetes mellitus (T2DM) attributable deaths per 100,000 people by country and year in women

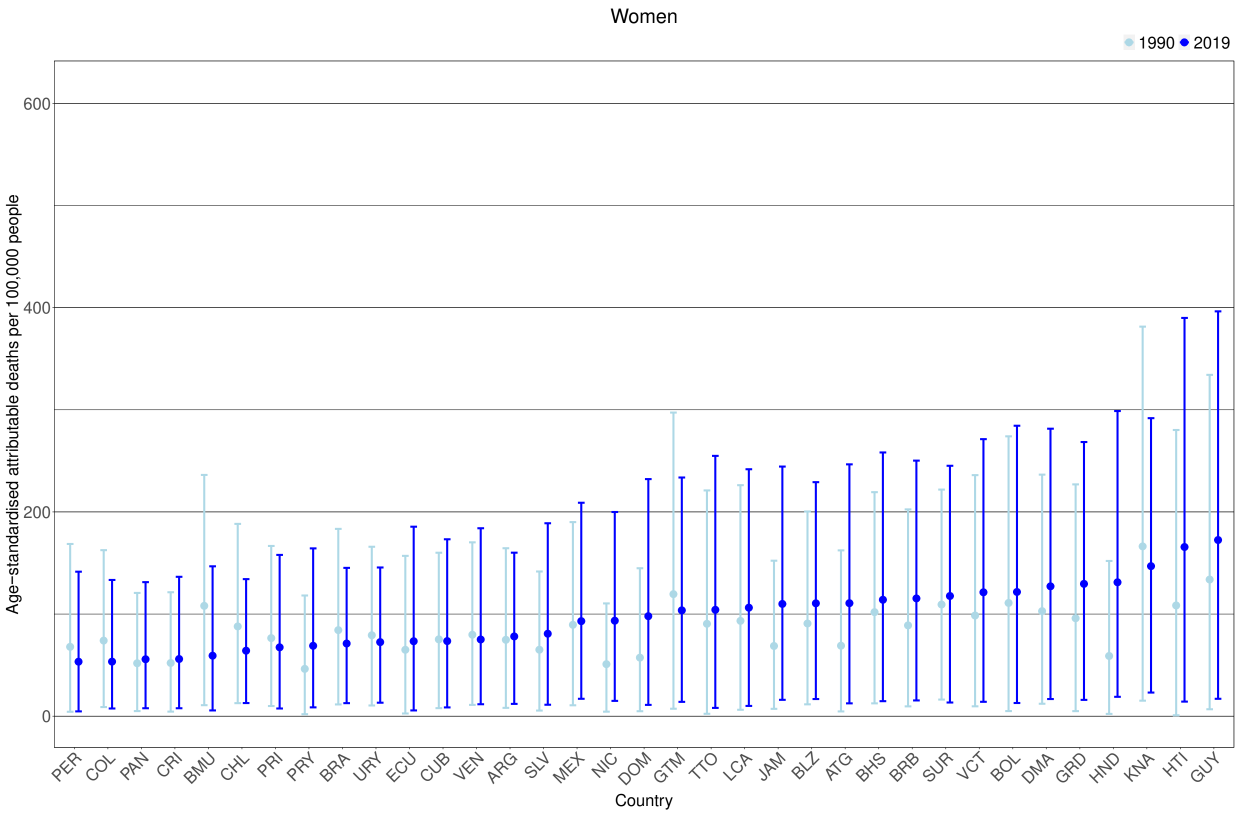

Supplementary figure 9: Correlations between the age-standardised T2DM-attributable death rates and economic metrics

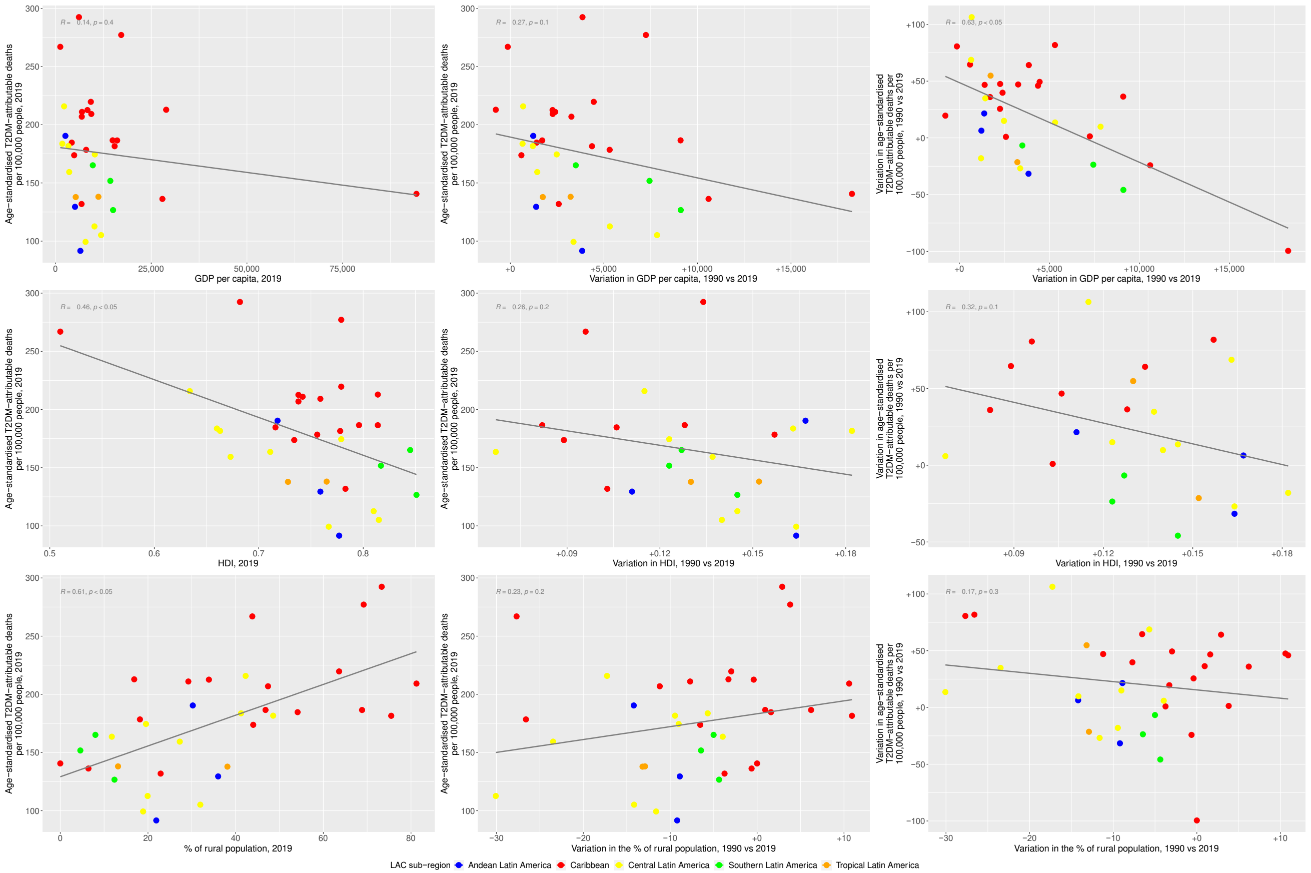

Supplement: Supplementary data [file bmjdrc-2021-002673supp001.pdf]
